# MMP-2 and MMP-9 in normal mucosa are independently associated with outcome of colorectal cancer patients

**DOI:** 10.1038/bjc.2012.80

**Published:** 2012-04-03

**Authors:** A M J Langers, H W Verspaget, L J A C Hawinkels, F J G M Kubben, W van Duijn, J J van der Reijden, J C H Hardwick, D W Hommes, C F M Sier

**Affiliations:** 1 Department of Gastroenterology-Hepatology, Leiden University Medical Centre, PO Box 9600, 2300RC, Leiden, The Netherlands; 2 Department of Molecular Cell Biology, Leiden University Medical Centre, Leiden, The Netherlands; 3 Department of Surgery, Leiden University Medical Centre, Leiden, The Netherlands

**Keywords:** matrix metalloproteinase, normal mucosa, carcinoma, SNP, survival, angiogenesis, ELISA

## Abstract

**Background:**

Upregulation of the matrix metalloproteinases MMP-2 and MMP-9 in various cancers has been associated with worse survival of the patients.

**Methods:**

We assessed MMP-2 and MMP-9 levels in normal colorectal mucosa from colorectal cancer patients in relation to the course of the disease.

**Results:**

A high protein expression of MMP-2 as well as MMP-9 in normal mucosa was found to be correlated with worse 5-year survival. The combination of both parameters was an even stronger prognostic factor. These protein levels were found not to be related to the corresponding single nucleotide polymorphisms of MMP-2 (−1306C>T) and MMP-9 (−1562C>T). Multivariate analyses indicated that the MMP-2 and MMP-9 levels in normal mucosa are prognostic for survival, independent of TNM classification.

**Conclusion:**

MMP-2 and MMP-9 levels in normal mucosa are indicative of the course of disease in colorectal cancer patients.

The gelatinases MMP-2 and MMP-9 are implicated in the process of colorectal cancer progression, angiogenesis and metastasis (Zucker and Vacirca, 2004). We previously reported that MMP-2 genotype and high levels of tumour MMP-2 are associated with a poor survival in colorectal cancer patients. For MMP-9, we found a bivalent correlation between MMP-9 expression and survival, that is, patients with either very low or very high MMP-9 tumour protein levels have worse survival compared with patients with intermediate MMP-9 expression (Langers *et al*, 2008). In contrast, little is known about the impact of MMP expression in normal mucosa of cancer patients. We hypothesised that the expression of MMP-2 and MMP-9 in normal mucosa of colorectal cancer patients could be relevant as well to the outcome in colorectal cancer. The aim of the present study was to evaluate the relation of the genotype and phenotype of MMP-2 and MMP-9 in normal appearing mucosa with outcome of patients with colorectal cancer.

## Materials and methods

### Patients and study design

Tumour tissue and normal appearing mucosa at a distance of 5–10 cm from the tumour was collected from 198 consecutive patients (85 female and 113 male) who underwent surgery for colorectal cancer between 1983 and 1991 at Leiden University Medical Centre. All patients from whom tissue was obtained and data collection was complete were included in this retrospective study of prospectively collected tissues. None of the patients received (neo−) adjuvant chemo- and/or radiotherapy. The surgical procedure consisted of removal of the tumour with *en bloc* resection of the lymph nodes. Tissue samples were snap frozen and stored at −70 °C until use. Macroscopic and microscopic parameters were obtained from the pathological reports, including TNM classification. Clinical data and follow-up information was available for a period of at least 5 years. The primary end point was survival at 5 years after surgery. Patient characteristics were as follows: 67% of patients was >65 years of age, TNM classification was stage 1 in 17%, stage 2 in 40%, stage 3 in 29% and stage 4 in 14% of the patients. The tumour was localised in the right hemicolon in 36% of the patients, 42% had left-sided cancer and in 22% the tumour was localised in the rectum. In 42% of the patients, the tumour was smaller than 5 cm in diameter and in 23% there was a mucinous component in the histology. Histological differentiation grade was poor in 13% of the patients, moderate in 70% and good in 17% of the patients. Parameters considered for inclusion in the multivariate analysis were: MMP-2 and MMP-9 in normal mucosa, tumour stage, age and gender. The study was performed according to the instructions and guidelines of the LUMC Medical Ethics Committee and in accordance with the Helsinki Declaration.

### Tissue preparation and protein concentration

Tissue homogenates were prepared in 0.1 M Tris-HCl (pH 7.5) with 0.1% (v/v) Tween 80 extraction buffer as previously described (Mulder *et al*, 1990). The protein concentration was determined (Lowry *et al*, 1951).

### Determination of MMP-2 and MMP-9 in tissue homogenates

Levels of MMP-2 and MMP-9 were determined in homogenates by previously described ELISAs (Gao *et al*, 2005). In short, polyclonal anti-MMP-2 or monoclonal anti-MMP-9 antibodies were used as catching antibody and appropriately diluted samples were incubated overnight at 4 °C. Immune-detection was performed using polyclonal rabbit anti-MMP-2 followed by biotin-labelled goat anti-rabbit-IgG for MMP-2 and biotin-labelled polyclonal anti-MMP-9 antibodies for MMP-9. After incubation with avidin-peroxidase, the chromogenic substrate 3,3′,5,5′-tetramethyl benzidine was added in the presence of hydrogen peroxide. The reaction was stopped with H_2_S0_4_ and the absorption was measured at 450 nm. The amount of MMP was calculated from parallel-incubated standard curves of MMP-2 or MMP-9 and expressed in ng per mg protein of the homogenate.

### Single nucleotide polymorphism (SNP) analysis

Genomic DNA was isolated from the tissues using the salting out method (Miller *et al*, 1988). The SNP analysis for MMP-2_−1306C>T_ and MMP-9_−1562C>T_ was performed by restriction fragment length polymorphism—polymerase chain reaction as described earlier (Kubben *et al*, 2006; Meijer *et al*, 2006) or by tetra primer ARMS PCR, involving four oligonucleotide primers but no restriction enzymes (Meijer *et al*, 2006). Genotype frequencies for MMP-2-1306C>T were CC: *n*=108: CT: *n*=79 and TT: *n*=11, and for MMP-9-1562C>T CC: *n*=139; CT: *n*=50 and TT: *n*=9.

### Statistical analyses

Statistical analyses were performed using SPSS 17.0. Statistical Package (SPSS Inc., Chicago, IL, USA). Expression differences between groups were calculated using the Mann–Whitney *U*-test. Log Rank statistics (LR) was used for optimal cut point analysis (Hothorn, 1994). Hardy–Weinberg analysis was performed using *χ*^2^ or Fisher's exact test to examine differences in the distribution of alleles and genotypes. Multivariate analyses were performed by adding every single MMP-related parameter to the dichotomised, prognosis-associated clinico-pathological parameters gender, age and TNM stage. Correlations between parameters were calculated according to Pearson's correlation test. *P*-values smaller than 0.05 were considered significant.

## Results

**Figure 1:**
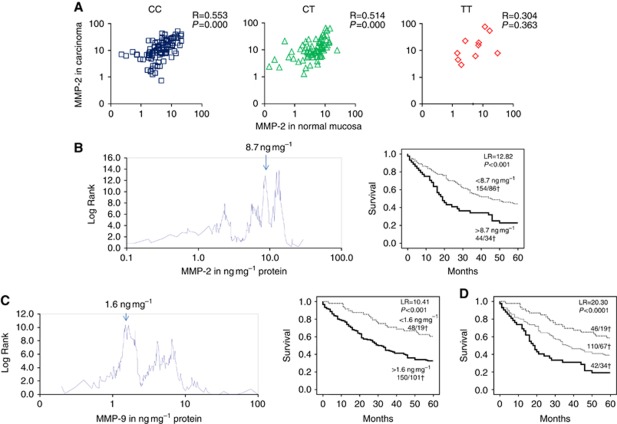
(**A**) Correlation between the tissue MMP-2 levels (in ng mg^−1^ protein) in normal mucosa and corresponding carcinoma tissue obtained from 198 colorectal cancer patients. The left panel shows the correlation in patients with a CC genotype, the middle panel in patients with a CT genotype and the right panel shows the correlation in patients with a TT genotype of the -1306CT polymorphism of MMP-2. Indicated are the Pearson's correlation coefficients for MMP-2 levels in normal mucosa and corresponding tumour and their corresponding *P*-value. (**B** and **C**) Optimal cutoff point analysis and corresponding Kaplan–Meier 5-year survival curves for dichotomised (high/low) levels of MMP-2 (**B**) and MMP-9 (**C**) in normal mucosa. The optimal cutoff points are indicated with arrows. In the survival curves, the total number of patients and the deceased patients (†) are indicated per subgroup. (**D**) Kaplan–Meier 5-year survival curves for combination of MMP-2 and MMP-9 in normal mucosa (both MMP-2 and MMP-9 high (lower, thick line), both MMP-2 and MMP-9 low (upper, dashed line), either MMP-2 high and MMP-9 low or MMP-2 low and MMP-9 high (intermediate, thin line); for cutoff points see **B** and **C**). The total number of patients and the deceased patients (†) are indicated per subgroup.

The median protein level of MMP-2 and MMP-9 in the normal mucosa of the 198 colorectal cancer patients was 4.8 ng mg^−1^ protein (range, 0–30.5) for MMP-2 and 3.2 ng mg^−1^ protein (range, 0.2–164.7) for MMP-9. As expected, MMP expression in normal mucosa was lower than in carcinoma tissue: 2-fold lower for MMP-2 (Figure 1A, median carcinomas 10.6 ng mg^−1^ protein) and 12-fold lower for MMP-9 (median carcinomas 36.7 ng mg^−1^ protein). The MMP-2 levels in the normal mucosa correlated significantly with the levels in cancer tissue (*R*=0.489, *P*⩽0.0001 for all genotypes together; results per genotype are shown in Figure 1A), whereas the MMP-9 levels did not (*R*=0.034, *P*=0.637). The highest levels of MMP-2 and MMP-9 were found in the mucosa from TNM stage IV patients, but the differences with and between the other stages were not statistically significant (data not shown). Optimal cutoff point analyses of mucosal MMP-2 and MMP-9 protein levels divided the patients in subgroups with significant differences in survival. High levels of MMP-2 (cutoff 8.7 ng mg^−1^ protein, LR 12.82, *P*<0.001, Figure 1B) or MMP-9 (cutoff 1.6 ng mg^−1^ protein, LR 10.41, *P*=0.001, Figure 1C) were associated with poorer 5-year survival. The combination of both mucosal MMP-based parameters appeared as a highly significant discriminator (LR 20.30, *P*<0.0001) for subdivision of patients into good, intermediate and poor survivors as shown in Figure 1D.

The genotypes of the MMP-2-1306C>T and MMP-9-1562C>T polymorphism were distributed according to the Hardy–Weinberg equilibrium (MMP-2 *χ*^2^=0.49, *P*=0.48; MMP-9 *χ*^2^=2.5, *P*=0.11) and there was no association between the different genotypes and the levels of MMP-2 and -9 in normal mucosa (data not shown).

Univariate Cox analysis confirmed the association of the tissue levels of MMP-2 and MMP-9 with survival (Table 1). Multivariate Cox analysis, including the clinical parameters gender, age and TNM stage of the tumour, showed both mucosal MMPs to be independent indicators of prognosis (hazard ratios >1.7, *P*=0.009). A separate multivariate analysis of the MMP-2/MMP-9 combination indicated a highly significant hazard ratio for patients with high mucosal levels of both MMPs.

## Discussion

In the present study, we found high protein levels of MMP-2 and MMP-9 in mucosa adjacent to colorectal cancer tissue to be indicative for the course of disease, that is, independently associated with a worse survival of the patients. Mucosa *immediately* adjacent to colon cancer (within 2 cm) shares histochemical, ultrastructural and biological features with the corresponding tumour and is referred to as transitional mucosa (Boland and Kim, 1987). But even at longer distances from the tumour, enhanced calcium levels and increased numbers of aberrant crypts are detected, indicating differences with mucosa from healthy controls (Edelstein *et al*, 1991; Pretlow *et al*, 1991). Some of these changes are clearly related to changes in the endothelium. ‘Normal’ mucosa adjacent to larger, more invasive tumours showed enhanced microvessel densities compared with less invasive tumours, which was associated with increased levels of the angiogenic factors vascular endothelial growth factor (VEGF), interleukin-8 and factor VIII-related antigen (Fox *et al*, 1998). These differences in the normal mucosa were not related to the survival of the patients. We have previously shown that low levels of the endothelium-derived serine proteinase tissue-type plasminogen activator (tPA) in normal appearing mucosa of colorectal and gastric cancer patients is also associated with poor prognosis (Ganesh *et al*, 1994; Ganesh *et al*, 1997). The correlation of high protein levels of MMP-2 and MMP-9 in mucosa adjacent to colorectal cancer tissue with worse survival, as we found, is probably also more, but not exclusively, related to endothelial cells in combination with epithelial cells and/or leukocytes. Previously we showed that MMP-2 in the intestinal mucosa of colorectal cancer patients is predominantly expressed in the submucosal extracellular matrix and MMP-9 in the mucosal macrophages and neutrophils (Gao *et al*, 2005). We hypothesised previously that the poor prognosis found for patients with very low tumour MMP-9 levels is possibly due to the lack of infiltrating leukocytes, which may possess anti-cancer effects (Langers *et al*, 2008). Diffuse staining of a subset of microvessels in tumour-adjacent mucosa of the oesophagus by CD105/endoglin, a neovascularisation marker, has been found to correlate statistically with worse overall survival of the patients (Bellone *et al*, 2007). Interestingly, we previously observed that MMP-9 is involved in the VEGF-mediated neo-angiogenesis in colorectal cancer and that MMP-mediated endoglin mobilisation is involved in the regulation of the angiogenic potential of endothelial cells in colorectal cancer (Hawinkels *et al*, 2008; Hawinkels *et al*, 2010). Angiogenesis in the adjacent mucosa might very well enhance the development of multi-focal tumours or regional metastasis, which could explain the correlation with survival.

We have previously shown that there is no association between SNPs of MMP-2 and MMP-9 and the amount of corresponding protein in colorectal carcinomas (Langers *et al*, 2008). But in a tumour environment, the final concentration of MMP-2 and -9 would depend on many different cell types, mechanisms and interactions. Therefore, one could speculate that an association between the SNP and the amount of MMP protein in normal mucosa would be more likely. T allele carriers of the MMP-2_−1306C>_T polymorphism have lower promoter activity due to disruption of an Sp-1-binding site (Price *et al*, 2001), while T allele carriers of the MMP-9_−1562C>T_ polymorphism have increased transcriptional activity due to preferential binding of a transcription suppressor protein to the C allele (Zhang *et al*, 1999). Although normal mucosa MMP-2 levels correlated significantly with their corresponding tumour levels, this was not restricted to or within a specific genotype. The tissue levels of MMP-9, either from normal mucosa or tumour, did not correlate and were also independent from the genotypic background. In a recent review about SNPs of MMPs and gastrointestinal cancer, we already emphasised that due to the complicated regulatory processes after transcription, SNPs are not very strong effectors of overall synthesis/presence of the corresponding MMP *in vivo* (Langers *et al*, 2011).

In conclusion, this study shows that increased MMP-2 and MMP-9 protein expression in normal mucosa at some distance of colorectal tumours is strongly related to the course of disease, that is, independently associated with a poor prognosis of colorectal cancer patients. The difference in mucosal expression of the gelatinases cannot be attributed to genotypic variations.

## Figures and Tables

**Table 1:** Univariate and multivariate Cox proportional hazard analysis for 5-year survival in normal appearing mucosa from 198 patients with colorectal cancer

		**Univariate**				**Multivariate**		
**Parameter**		***N***	**HR**	**CI 95%**	***P***	**HR**	**CI 95%**	***P***
Gender	F-M	85–113	1.195	0.830–1.719	0.339	1.164	0.808–1.677	0.414
Age	<65 years>	66–132	1.861	1.230–2.815	**0.003**	2.003	1.322–3.035	**0.005**
TNM	I+II-III+IV	113–85	2.972	2.056–4.297	**0.000**	3.083	2.129–4.465	**0.000**
								
*MMP mucosal*								
MMP-2	<8.7>	154–44	1.898	1.273–2.828	**0.002**	1.718	1.145–2.578	**0.009**
MMP-9	<1.6>	48–150	2.323	1.421–3.796	**0.001**	1.948	1.184–3.204	**0.009**
								
MMP-2/MMP-9 combination^a^	<,<	46	Ref	—	—	Ref	—	—
	Rest	110	1.839	1.105–3.063	**0.019**	1.582	0.946–2.645	0.080
	>,>	42	3.310	1.882–5.821	**0.000**	2.638	1.486–4.683	**0.001**

Abbreviations: CI=confidence interval; F-M=female-male; HR=hazard ratio; MMP=matrix metalloproteinase; Ref=reference group.

a In the multivariate analysis for the MMP-2/MMP-9 combination, the separate variables MMP-2 and MMP-9 protein were not included.

<,<=MMP-2<8.7 ng mg^−1^ protein and MMP-9<1.6 ng mg^−1^ protein; >,>=MMP-2>8.7 ng mg^−1^ protein and MMP-9>1.6 ng mg^−1^ protein.

Multivariate analysis was performed by adding every single MMP-related parameter to the dichotomised, prognosis-associated clinico-pathological parameters gender, age and TNM stage. Statistically significant values are given bold.

## References

[bib1] Bellone G, Solerio D, Chiusa L, Brondino G, Carbone A, Prati A, Scirelli T, Camandona M, Palestro G, Dei PM (2007) Transforming growth factor-beta binding receptor endoglin (CD105) expression in esophageal cancer and in adjacent nontumorous esophagus as prognostic predictor of recurrence. Ann Surg Oncol 14(11): 3232–3242 17682823 10.1245/s10434-007-9528-z

[bib2] Boland CR, Kim YS (1987) Transitional mucosa of the colon and tumor growth factors. Med Hypotheses 22(3): 237–243 3473277 10.1016/0306-9877(87)90189-7

[bib3] Edelstein PS, Thompson SM, Davies RJ (1991) Altered intracellular calcium regulation in human colorectal cancers and in “normal” adjacent mucosa. Cancer Res 51(16): 4492–4494 1868472

[bib4] Fox SH, Whalen GF, Sanders MM, Burleson JA, Jennings K, Kurtzman S, Kreutzer D (1998) Angiogenesis in normal tissue adjacent to colon cancer. J Surg Oncol 69(4): 230–234 9881940 10.1002/(sici)1096-9098(199812)69:4<230::aid-jso7>3.0.co;2-q

[bib5] Ganesh S, Sier CF, Griffioen G, Vloedgraven HJ, De Boer A, Welvaart K, van de Velde CJ, van Krieken JH, Verheijen JH, Lamers CB (1994) Prognostic relevance of plasminogen activators and their inhibitors in colorectal cancer. Cancer Res 54(15): 4065–4071 8033138

[bib6] Ganesh S, Sier CF, Heerding MM, van Krieken JH, Griffioen G, Welvaart K, van de Velde CJ, Verheijen JH, Lamers CB, Verspaget HW (1997) Contribution of plasminogen activators and their inhibitors to the survival prognosis of patients with Dukes’ stage B and C colorectal cancer. Br J Cancer 75(12): 1793–1801 9192984 10.1038/bjc.1997.306 PMC2223607

[bib7] Gao Q, Meijer MJ, Kubben FJ, Sier CF, Kruidenier L, van DW, van den Berg M, van Hogezand RA, Lamers CB, Verspaget HW (2005) Expression of matrix metalloproteinases-2 and -9 in intestinal tissue of patients with inflammatory bowel diseases. Dig Liver Dis 37(8): 584–592 15869913 10.1016/j.dld.2005.02.011

[bib8] Hawinkels LJ, Kuiper P, Wiercinska E, Verspaget HW, Liu Z, Pardali E, Sier CF, Ten DP (2010) Matrix metalloproteinase-14 (MT1-MMP)-mediated endoglin shedding inhibits tumor angiogenesis. Cancer Res 70(10): 4141–4150 20424116 10.1158/0008-5472.CAN-09-4466

[bib9] Hawinkels LJ, Zuidwijk K, Verspaget HW, de Jonge-Muller ES, van Duin W, Ferreira V, Fontijn RD, David G, Hommes DW, Lamers CB, Sier CF (2008) VEGF release by MMP-9 mediated heparan sulphate cleavage induces colorectal cancer angiogenesis. Eur J Cancer 44(13): 1904–1913 18691882 10.1016/j.ejca.2008.06.031

[bib10] Hothorn L (1994) Biostatistical analysis of the micronucleus mutagenicity assay based on the assumption of a mixing distribution. Environ Health Perspect 102(Suppl 1): 121–125 10.1289/ehp.94102s1121 PMC1566887 8187700

[bib11] Kubben FJ, Sier CF, Meijer MJ, van den Berg M, Van der Reijden JJ, Griffioen G, Van de Velde CJ, Lamers CB, Verspaget HW (2006) Clinical impact of MMP and TIMP gene polymorphisms in gastric cancer. Br J Cancer 95: 744–751 16940985 10.1038/sj.bjc.6603307 PMC2360506

[bib12] Langers AM, Sier CF, Hawinkels LJ, Kubben FJ, van DW, van der Reijden JJ, Lamers CB, Hommes DW, Verspaget HW (2008) MMP-2 geno-phenotype is prognostic for colorectal cancer survival, whereas MMP-9 is not. Br J Cancer 98(11): 1820–1823 18506186 10.1038/sj.bjc.6604380 PMC2410128

[bib13] Langers AM, Verspaget HW, Hommes DW, Sier CF (2011) Single-nucleotide polymorphisms of matrix metalloproteinases and their inhibitors in gastrointestinal cancer. World J Gastrointest Oncol 3(6): 79–98 21731908 10.4251/wjgo.v3.i6.79 PMC3124635

[bib14] Lowry OH, Rosebrough NJ, Farr AL, Randall RJ (1951) Protein measurement with the Folin phenol reagent. J Biol Chem 193(1): 265–275 14907713

[bib15] Meijer MJ, Mieremet-Ooms MA, Van Duijn W, van der Zon AM, Hanemaaijer R, Verheijen JH, Van Hogezand RA, Lamers CB, Verspaget HW (2006) Effect of the anti-tumor necrosis factor-alpha antibody infliximab on the *ex vivo* mucosal matrix metalloproteinase-proteolytic phenotype in inflammatory bowel disease. Inflamm Bowel Dis 13: 200–210 10.1002/ibd.20051 17206679

[bib16] Miller SA, Dykes DD, Polesky HF (1988) A simple salting out procedure for extracting DNA from human nucleated cells. Nucleic Acids Res 16(3): 1215 3344216 10.1093/nar/16.3.1215 PMC334765

[bib17] Mulder TP, Verspaget HW, Janssens AR, De Bruin PA, Griffioen G, Lamers CB (1990) Neoplasia-related changes of two copper (Cu)/zinc (Zn) proteins in the human colon. Free Radic Biol Med 9(6): 501–506 2079229 10.1016/0891-5849(90)90128-6

[bib18] Pretlow TP, Barrow BJ, Ashton WS, O′Riordan MA, Pretlow TG, Jurcisek JA, Stellato TA (1991) Aberrant crypts: putative preneoplastic foci in human colonic mucosa. Cancer Res 51(5): 1564–1567 1997197

[bib19] Price SJ, Greaves DR, Watkins H (2001) Identification of novel, functional genetic variants in the human matrix metalloproteinase-2 gene: role of Sp1 in allele-specific transcriptional regulation. J Biol Chem 276(10): 7549–7558 11114309 10.1074/jbc.M010242200

[bib20] Zhang B, Ye S, Herrmann SM, Eriksson P, de Maat M, Evans A, Arveiler D, Luc G, Cambien F, Hamsten A, Watkins H, Henney AM (1999) Functional polymorphism in the regulatory region of gelatinase B gene in relation to severity of coronary atherosclerosis. Circulation 99(14): 1788–1794 10199873 10.1161/01.cir.99.14.1788

[bib21] Zucker S, Vacirca J (2004) Role of matrix metalloproteinases (MMPs) in colorectal cancer. Cancer Metastasis Rev 23(1-2): 101–117 15000152 10.1023/a:1025867130437

